# The value of psychological treatment for borderline personality disorder: Systematic review and cost offset analysis of economic evaluations

**DOI:** 10.1371/journal.pone.0171592

**Published:** 2017-03-01

**Authors:** Denise Meuldijk, Alexandra McCarthy, Marianne E. Bourke, Brin F. S. Grenyer

**Affiliations:** Illawarra Health and Medical Research Institute and School of Psychology, University of Wollongong, Wollongong, New South Wales, Australia; Central Institute of Mental Health, GERMANY

## Abstract

**Aim:**

Borderline Personality Disorder (BPD) is a common mental health condition with high patterns of service utilisation of inpatient and community treatment. Over the past five years there has been significant growth in research with economic data, making this systematic review a timely update.

**Methods:**

Empirical studies written in English or German, published up to December 2015, and cited in major electronic databases were examined using the PRISMA systematic review method. Papers were included that had one of the following: data related to cost of BPD to society, the individual, the carer or families; cost benefits of interventions. Reported cost data were inflated to the year 2015 and converted into US- dollars (USD $) using purchasing power parities.

**Results:**

We identified 30 economic evaluations providing cost data related to interventions for BPD across 134,136 patients. The methodological quality was good, almost all studies fulfilled ≥ 50% of the quality criteria. The mean cost saving for treating BPD with evidence-based psychotherapy across studies was USD $2,987.82 per patient per year. A further mean weighted reduction of USD $1,551 per patient per year (range $83 - $29,392) was found compared to treatment as usual. Evidence-based psychological treatment was both less expensive as well as more effective, despite considerable differences in health cost arrangements between individual studies and countries. Where it was able to be calculated, a significant difference in cost-savings between different types of evidence-based psychotherapies was found.

**Discussion:**

Individuals with BPD consistently demonstrate high patterns of service utilization and therefore high costs. The findings of this review present a strong argument in favour of prioritizing BPD treatments in reimbursement decisions, both for the affected individual and the family. The provision of evidence based treatment, irrespective of the type of psychological treatment, may lead to widespread reductions in healthcare costs.

## Introduction

In a budget constrained health care system, the cost and benefits of psychological interventions are increasingly of interest as decisions regarding resource allocation have forced individuals to re-examine the cost effectiveness of psychological treatments [1,2]. In particular clinicians are increasingly faced with numerous challenges surrounding decisions regarding treatment options which need to be both ethically informed and based on available research findings. Concurrent with the debate on health care reform, and the relative dearth of evidence-based treatment for Personality Disorders (PD), clinicians have become increasingly interested in evaluating the cost-effectiveness of psychological treatments [3]. Attention to the cost-benefit analysis is crucial in weighing the benefits versus disadvantages of specific interventions and treatment approaches [4], clearly defining the method, duration and outcomes of interventions. Managed care providers demand that treatments have demonstrated efficacy, minimize costs, and reduce future utilization of expensive resources.

Previous research in the field has highlighted that individuals suffering Borderline Personality Disorder (BPD) pose a high economic burden on society due to their extensive use of treatment services [5–7]. This is understandable as BPD is present in 1–2% of the population, 10% of psychiatric outpatients and between 15% and 25% of inpatients [8–10]. Additionally patients with BPD present to services at a high risk of self-harm, with up to 10% of psychiatric outpatients committing suicide, a rate almost 50 times higher than the general population [11]. The widespread economic impacts of the condition is aggravated by the fact that individuals suffering BPD typically need several repeated treatments, including urgent interventions in emergency departments and seek help repeatedly and simultaneously from multiple sources [12,13].

Within mental health settings, Sansone and colleagues (2011) [6] found that patients with BPD symptomology appear to have a significantly higher turnover with primary care physicians and see a greater number of specialists than patients without these symptoms. Additionally, Bender and colleagues (2001) [5] found that compared to individuals with major depression, those with BPD were significantly more likely to use most types of psychiatric treatment. Finally, in an Australian community sample, Jackson and Burgess (2004) [14] found that individuals with BPD were more likely than other individuals to seek psychiatric or psychological consultations. Given the seemingly consistent pattern of high utilization of mental health services among individuals with BPD, these findings may reflect the underlying psychological processes of the disorder as well as a general pattern of demand for healthcare services [15].

Treatment guidelines for BPD support psychotherapy in the community as the treatment of choice [16]. Studies of different psychological therapies such as mentalization based treatment, transference focused therapy and dialectical behaviour therapy, have demonstrated positive outcomes in relation to symptomology and levels of service use [9]. However, due to the nature of BPD symptoms, the identification of psychotherapies and utilisation of interventions that are cost-effective with this population is of considerable importance. While many factors influence the likelihood that a treatment is effective, one of the primary factors has become the affordability of treatment. That is, the pure monetary value one saves or loses by investing in one treatment compared to another. Brazier and colleagues (2006) [17] completed a systematic review and a preliminary economic evaluation of available therapies for BPD patients [17]. In 2014, Brettschneider and colleagues [18] performed a systematic literature review of 15 existing economic evaluations of treatments for BPD up to 2012, but the economic evaluations reviewed used different comparators to define costs, which made comparability of the data difficult and the findings not sufficient to draw robust conclusions for all treatments. There has been no updated review over the past 5 years despite a surge in economic data available.

Therefore the present study aims to systematically review and synthesize the literature on this topic and provide an updated overview (i.e. up to December 2015). We are specifically focused at looking at the cost-benefit of recognised evaluated treatments, as the results can be used to gain insight into how much society is spending on BPD, and potentially how much can be saved if effective therapy is offered. The information can be helpful in setting priorities for health care efficiency research as high societal costs present a strong argument in favour of prioritizing BPD treatments in reimbursement decisions, in terms of health insurance benefits for affected individuals and funding grants to provide accessible and effective treatment services.

## Materials and methods

### Protocol and registration

This review followed the Preferred Reporting Items for Systematic Reviews and Meta-Analysis (PRISMA) Statement for Reporting Systematic Reviews [19] and additional guidelines for conducting and reporting systematic reviews [20]. The protocol was registered by the International Prospective Register of Systematic Reviews (registration number: CRD42016037305).

### Data sources

Literature was searched for relevant articles published up to December 2015 using the following online databases: Psychological and Behavioural Sciences, PsychInfo, Scopus, Medline, Web of Science and PubMed. Search terms used for each database included the following: Borderline Personality Disorder OR Personality Disorder AND cost OR cost analysis OR cost benefit OR cost effectiveness OR economic analysis OR (burden AND (society OR community OR individual OR carer OR carers)). This search identified papers directly related to the research questions.

### Study selection

The eligibility of the studies was assessed in two steps. First titles and abstracts were screened. Subsequently, articles considered as relevant were obtained and the full text was screened. All original studies reporting cost or cost-effectiveness data of BPD were included.

Studies were eligible for further consideration in this review if they related to the costs or benefits of interventions for BPD (specifically cost or benefit to the individual, carer or family, or society either through direct (e.g. medical) or indirect (e.g. loss of productivity) costs or benefits. Studies were excluded if they were not in English or German, or did not publish original empirical data (e.g. newspaper articles, reviews, commentaries and editorials). Records identified by the searching process were screened by two authors (DM, AM) and checked by another (BG).

### Data extraction and risk of bias

Two authors (DM and AM) extracted data from the included studies, which was then independently reviewed by another author (BG). This author was blind to prestige factors, including authors, institutions, journal titles, and publishers. The authors compared their findings and discrepancies were discussed and resolved to reach a consensus.

Data extracted included: *country of publication*, *cost year*, *study sample characteristics (number of participants*, *age*, *gender*, *types of treatment compared)*, *cost (per population and per patient)*, *and if stated*, *type of economic evaluation*.

Two categories of costs can be distinguished: *direct* and *indirect costs;* [21,22]. *Direct costs* include all treatment costs that arise directly from medical care. These include items such as the cost of psychiatric inpatient and outpatient care, psychological, rehabilitation, medication, or emergency room treatments. *Indirect costs* cover all costs that occur secondary to the disease. These include items such as production losses and sickness benefit payments due to days absent at work, as well as years lost due to mortality. In addition, the total costs (either indirect or direct) associated with the delivery of the intervention (intervention costs) were also presented.

For the purpose of this review, type of economic evaluation [21], was described and defined as follows:

1. *Full economic evaluations*. A full economic evaluation does not only compare costs of at least two interventions but also their consequences (outcomes, effects) [21]. Effects can be measured in natural units (life years gained, parasuicide events avoided, artificial units (quality-adjusted life years [QALY] or disability-adjusted life years [DALY]) or monetary units measured by techniques like willingness-to-pay experiments. Depending on the effect measure employed, full economic evaluations are called cost-effectiveness analyses (natural units), cost-utility analyses (utility measures) or cost-benefit analyses (outcomes valued monetarily).
2. *Partial economic evaluations*. Health economic studies considered to be partial economic evaluations do not make explicit comparisons between alternative interventions in terms of both costs (resource use) and consequences (effects). In partial economic evaluations only the costs of at least two alternatives are compared (i.e. cost analyses, cost-description studies and cost-outcome descriptions). Partial evaluations can be useful in that they can provide elements of information for a full evaluation and help answer questions not related to efficiency.

Whilst the methodologies of full economic evaluations and partical economic evaluations are distinct from each other, both types of economic evaluations were included for the purposes of our review. Debate exists as to whether the information derived from partial economic evaluations are of the same scientific value and usefulness as the information derived by full economic evaluations, nevertheless, including both types of economic evaluations will contribute useful evidence to an understanding of economic aspects of interventions.

### Quality assessment

Quality assessment of the studies included was performed by means of the Consensus on Health Economic Criteria (CHEC) checklist. The CHEC-list has been developed using a Delphi method (three Delphi rounds; 23 international experts). The CHEC-list comprehends 19 criteria formulated as a question for answering either by "yes" or "no" [23].

The results of the quality assessment are displayed as percentage of studies fulfilling each criterion.

### Cost-offset analysis

The focus of this review is on costing issues associated with the provision of interventions for the treatment of BPD, including costs, cost-effectiveness and cost offset (i.e., a reduction in health care costs attributable to the intervention provided). Cost offsets originate from the use of mental health services and related costs before, during and after (if applicable) a specific intervention. Interventions included were recognised psychological treatments that have separately been shown to have clinical effectiveness and therefore an evidence-base [9].Therefore data was extracted based on cost alone, without further consideration of clinical effectiveness as this had been separately established and is outside the scope of this review.

#### Cost offset PT

The cost offset of psychotherapeutic interventions (PT) (Cost offset PT) was calculated by subtracting the total costs *after* the intervention provided from the total costs before the *start* of the intervention (i.e. pre-post differences in healthcare costs).

#### Cost offset PT vs. TAU

The cost offset of a psychotherapeutic intervention PT vs. TAU (Cost offset PT vs. TAU) represents the difference in total costs after the intervention is provided compared to cost related to the provision of treatment as usual (TAU) (i.e. post- difference in healthcare cost related to PT vs. TAU).

In order to assess the costs and cost offsets, and to present a financial evaluation, we applied present-day financial costing standards to the data. Cost data were inflated and converted to 2015 US-$ purchasing power parities (PPP) to ensure comparability of the data [24].

If an included study did not report the cost year, the year in which the study was accepted for publication was used as a proxy. To further ensure comparability of the data, costs per patient were calculated if costs data related to groups or a population. Subsequently, if the time horizon chosen in the study was less or more than one year, costs were converted to one year costs.

Due to the variation in sample size, means weighted for sample size were calculated and reported when presenting the overall cost offset of PT and the overall cost offset of PT vs TAU of all the economic evaluations identified in this review, and where appropriate, compared using independent samples t test.

## Results

### Search results

The search of electronic databases resulted in the identification of 4660 studies (4380 with duplicates removed). An additional 25 studies were identified through cross referencing and consultation with experts, yielding 4405 potentially relevant articles.

Of these, 4220 were excluded as their titles and/or abstracts clearly indicated that they did not meet the inclusion criteria.

Of the remaining 185 articles, 137 were excluded because primary focus was not BPD (n = 39), the articles were a secondary review without original data (n = 12), or the articles contained no cost or benefit data (n = 86).

**Fig 1:**
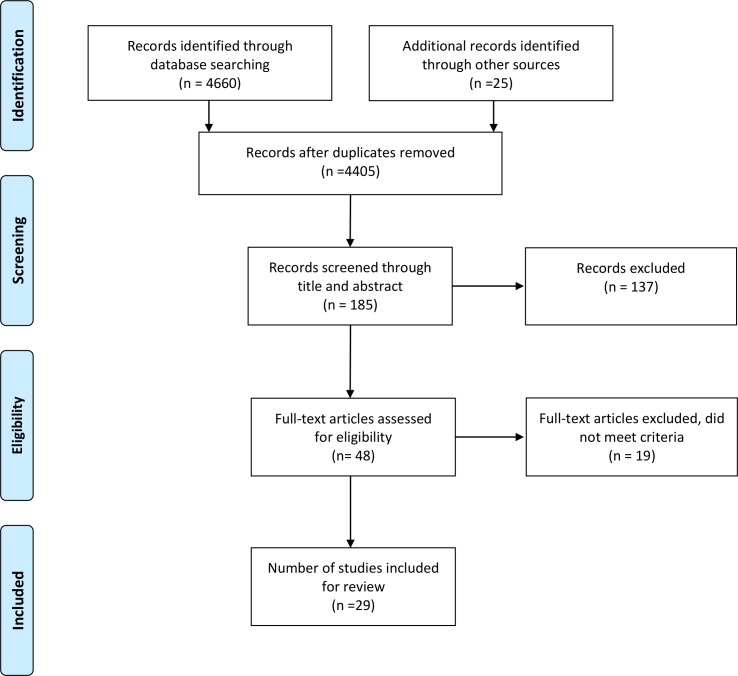
PRISMA Flowchart for the selection of studies included in the systematic review.

Full text screening was performed for the remaining 48 articles. 19 articles were excluded of which 12 did not report on cost data, four were not mainly focused on BPD and three were review articles; not containing original data. Finally, 29 articles (30 evaluations) were considered in this review (see **Fig 1** for PRISMA Flowchart).

### Characteristics of studies included

The characteristics of the included studies are summarized in **Table 1**.

**Table 1:** Summary of the cost differences in the economic evaluations reviewed (indirect/direct + intervention costs calculated for 2015, per patient, time horizon as stated).

Study	Currency	Study Type	Sample Size	Mean age	Gender	Time horizon	Treatment		Direct Costs^a^	Indirect Costs^a^	Intervention Costs^a^	Cost Offset PT ^b^	Cost Offset PT vs. TAU ^b^
Amner, 2012 [28]	GBP	Partial	21	36.2	81% F	1 year	DBT	pre	8,715		8,670	1,829	
								post	6,885				
Bateman & Fonagy, 2003^c^ [25]	USD	Partial	41	31.8	58% F	3 years	MBT	pre	2,633		1,599	811	283
								post	187				
							TAU	pre	3,564		2,100		
								post	1,050				
Berrino et al. 2011^c^ [48]	CHF	Full	200	32.1	85% F	3 months	CI	post	11,775				11,988
							TAU	post	14,773				
Borschmann et al., 2013 [49]	GBP	Full	88	35.8	81% F	6 months	CP	post	6,459		178		786
							TAU	post	6,852				
Brazier et al. 2006 [17] based on	GBP	Full	24	22	79% F	1 year	DBT	post	22,320		14,705	7,432	
Turner et al. 2000^c^ [32]							CCT	post	29,760		13,214		
Brazier et al. 2006 [17] based on	GBP	Full	44	N.A.	N.A.	1 year	DBT	post	33,510		27,834		2578
on Linehan et al. 1991^c^ [40]							TAU	post	36,088		16,995		
Brazier et al. 2006 [17] based on	GBP	Full	58	37.5	100% F	1 year	DBT	post	20,911		14,392		-869
van den Bosch et al. 2002^c^ [41]							TAU	post	20,042		7,270		
Brazier et al. 2006 [17] based on	GBP	Full	28	35	100% F	1 year	DBT	post	29,271		21,112		-10,771
Koons et al. 2001^c^ [42]							TAU	post	18,504		10,249		
Brazier et al. 2006 [17] based on	GBP	Full	38	31.8	58% F	1 year	MBT	post	27,858				-661
Bateman et al. 1999^c^ [47]							TAU	post	27,198				
Brazier et al. 2006 [17] based on	GBP	Full	480	32	68% F	1 year	MACT	post	12,716				-2677
Tyrer et al. 2003^c^ [50]							TAU	post	10,038				
Davidson et al. 2010^c^ [51]	GBP	Full	76	31.9	84% F	6 years	CBT-PD	post	186				84
							TAU	post	691				
Goodman et al., 2011^c^ [27]	USD	Partial	233	51.32	95% F	1 year	N.A.	post			17,3707		
Grazia Turri & Andreatta, 2014 [36]	GBP	Partial	1	N.A.	N.A.	9 years	LT-P	pre	3,165		2,048	271	
								post	731				
Hall et al. 2001 [54]	AUD	Partial	30	29.4	63% F	1 year	CM	pre	31,340		5,322	22,366	
								post	8,974				
Heard, 2000 (study one) [33]	USD	Full	44	N.A.	N.A.	1 year	DBT	post	637				640
							TAU	post	1,277				
Heard, 2000 (study two) [33]	USD	Full	38	N.A.	N.A.	1 year	DBT	post	638			456	
							SCP	post	1,094				
Kvarstein et al., 2013 [37]	EUR	Full	107	31	76% F	3 years	SDC	pre	825		2,389	586	
								post	776				
							OPC	pre	1,373		1,953	11,713	
								post	398				
Meyers et al., 2014^c^ [29]	USD	Partial	41	47.1	46% F	1,5 year	DBT	pre	33,323		8,453	3,333	
								post	29,990				
Palmer et al., 2006 [26]	GBP	Full	106	31.9	84% F	2 years	CBT	pre	138		37	-99	83
								post	336				
							TAU	pre	215				
								post	501				
Pasieczny & Connor, 2011 [43]	AUD	Full	90	33.58	93% F	3 years	DBT	post	24,920				2,092
							TAU	post	25,966				
Perseius et al., 2004 [30]	SEK	Partial	22	N.A.	N.A.	2,5 years	DBT	pre	11,0168		10,073	56,024	
								post	26,132				
Prendergast &	AUD	Full	11	36.35	100% F	1 year	DBT	post					11,603
McCausland,2007^c^ [44]							TAU						
Priebe et al., 2012 [45]	GBP	Full	80	32.2	100% F	1 year	DBT	post	173	34			-61
							TAU	post	114	31			
Richter et al., 2014^c^ [46]	EUR	Full	31	33.3	94% F	12 weeks	DBT	post					29,392
Stevenson & Meares, 1999 [34]	AUD	Partial	30	29.4	63% F	1 year	CM	pre	25,676		14,633	24,122	
								post	1,554				
Van Asselt et al. 2007 [3]	EUR	Full	131900	30.5	92% F	1 year	N.A.	pre	10,081	10,850			
Van Asselt et al., 2008^a^ [39]	EUR	Full	86	30.6	93% F	5 year	SFT	post	33,795	13,273		2,825	
							TFP	post	44,116	14,254			
Van Asselt et al., 2008^b^ [38]	EUR	Partial	86	30.6	93% F	4 years	SFT	post		263^d^		4	
							TFP	post		278^d^			
Wagner et al., 2013 [53]	EUR	Partial	55	30.2	93% F	1 year	DBT	pre	22,397	11,096			
Wagner et al., 2014 [31]	EUR	Partial	47	30.1	92% F	3 year	DBT	pre	505	238	497	352	
								post	174	217			
**Total**			**134136**	**30.56**^e^	**89% F**^e^								

^a^ Foreign currencies are converted to US dollars, per patient. Then costs were inflated to 2015 US dollars.

^b^Cost offsets were calculated for 2015 US dollars costs, per patient, per year. PT = Psychotherapy; TAU = Treatment As Usual.

^c^Cost year was not reported in the study, year of publication was used as proxy.

^d^Average of calculated bootstrapped productivity costs according to the Friction Cost (FC) Method and the Human Capital (HC)- method (i.e. HC–limited and HC -extended Method).

^e^ Weighted averages

**Cost Offset PT** = Difference in pre and post total (indirect + direct) costs for PT: **Cost Offset PT vs. TAU** = Difference in post total (indirect + direct) costs for PT vs. TAU.

**DBT:** Dialectical Behaviour Therapy; **CBT-PD**: Cognitive-Behavioural Therapy for personality disorders, **CBT:** Cognitive Behavioral Therapy in addition to TAU; **CCT**: Client Centered Therapy control condition; **CI**: Crisis Intervention at the General Hospital; **CP**: Joint Crisis Plan + Treatment As Usual; **CM:** Conversational Model; **LT-P**: Long Term Psychoanalytic Psychotherapy; **MACT**: Manual-Assisted Cognitive Behavioural Therapy; **MBT**: Mentalization Based Therapy; **OPC**: Outpatient Individual Psychotherapy; **SCP**: Stable Psychotherapy in Community; **SDC**: Individual and group therapy; **SFT**: Schema-Focused Therapy; **TFP**: Transference-Focused Therapy.

AUD: Australian Dollar; CHF: Swiss Franc; EUR: Euros; GBP: Pound Sterling; SEK: Swedish Krona; USD: United States Dollar.

N.A.: Not Available; F: female.

Of the total 30 evaluations (29 articles) considered in this review, 19 evaluations were full evaluations and 11 evaluations were partial economic evaluations. Twelve evaluations were conducted in the United Kingdom (UK), five in the United States of America, four each in the Netherlands and in Australia, three in Germany and one each in Norway and Switzerland.

The majority of the evaluations employed the societal perspective. The time horizon of the evaluations ranged from 12 weeks to nine years. Most evaluations included less than 100 patients (n = 24), some even less than 50 (n = 16). We included five economic evaluations with a study population of n > 100 patients. The 30 evaluations bring the total number of patients in this review to n = 134,136.

In all evaluations the majority of patients were female; in most evaluations (n = 16) the proportion of female patients was larger than 80%. The overall weighted percent of female patients across the studies included was 89%.

The mean age of populations ranged from 22 years to 51 years. The weighted average age was 30.65 years across the 30 studies included.

### Treatments compared

Regarding treatment comparisons, the studies generally fell into two following categories: 1) studies that examine (one or two competing) psychotherapeutic interventions or 2) studies examining psychotherapeutic interventions versus Treatment As Usual (TAU). A few studies included both categories [25,26]. In addition to the pre-post analysis comparing cost pre- and post intervention the study by Bateman & Fonagy (2003) [25] and the study by Palmer and colleagues (2006) [26] also examined the pre- and post- intervention costs in comparison to the provision of TAU. In two evaluations [3,27], the examined intervention was clarified and no comparison of treatments was made.

#### Studies comparing psychotherapeutic interventions

A total of fifteen evaluations examined the use of mental health services and related costs associated with the provision of a psychological treatment (i.e. other than TAU) in BPD patients.

Of these, seven focused on the costs solely related to the provision of DBT [28–33] Of latter two studies DBT was compared to an alternative psychotherapeutic intervention [32,33]. Turner and colleagues (2000) [32] reported on a comparison of DBT with a client-centered therapy control condition (CCT) whereas Heard and colleagues (2000) [33] conducted a comparison between a stable psychotherapy in community (SCP) and DBT.

Other psychotherapeutic interventions examined in these fifteen evaluations are: Conversational Model (CM) [34,35]; Long term psychoanalytic psychotherapy (LT-P) [36]; Kvarstein and colleagues (2013) [37] provided the pre- and post- costs associated with Individual and group therapy giving in a step-down condition (SDC) and Outpatient Individual Psychotherapy (OPC). In two evaluations Schema Focused Therapy (SFT) and Transference Focused Psychotherapy (TFP) were the competing psychotherapeutic interventions [38,39]. Mentalization Based Therapy (MBT) was examined in the study by Bateman & Fonagy (2003) [25] whereas Palmer and colleagues (2006) [26] provided the cost associated with the provision of Cognitive Behavioural Therapy (CBT) in addition to TAU. See **Table 1**.

#### Studies comparing psychotherapeutic interventions versus treatment as usual

Fifteen evaluations studied a psychotherapeutic intervention against treatment as usual (TAU). Of these, eight evaluated dialectical behaviour therapy (DBT) with TAU [33,40–46]. Two studies evaluated Mentalization based therapy (MBT) with TAU [25,47]. The following psychotherapeutic interventions were investigated by one evaluation each, using TAU for comparison: Crisis intervention at the general hospital (CI) [48], Joint crisis plan plus treatment as usual (CP) [49], manual assisted cognitive behavioural therapy (MACT) [50], Cognitive behavioural therapy for personality disorders (CBT-PD) [51] and Cognitive Behavioural Therapy (CBT) in addition to TAU [26]. In all of these studies, TAU was considered as standard of care for all patients and could involve a range of therapeutic options.

#### Methodological quality of identified studies

The results of the assessment of methodological quality are presented in Table 2. Each criteria of the quality assessment was fulfilled by the majority of the studies (≥ 50% of the studies). Criteria 14 and criteria 19 were fulfilled by 12 (43%) of the 30 studies included. None of the included studies fulfilled *all* of the quality criteria (Table 2). However, in the context of this review, the description of model details and cost data was suboptimal in most of the studies, with Pasciezny and Conner (2011) [43] being the exception (8 quality criteria were fulfilled).

**Table 2:** Economic studies meeting the criteria for methodological quality on the CHEC-list (Evers, Goossens, de Vet, van Tulder, & Ament, 2005).

Criteria	Yes	Proportion of studies fulfilled criteria
1. Is the study population clearly described?	[3–25–26–27–28–29–30–31–33–34–36–37–38–39–42–43–44–45–46–47–48–49–50–51–53–54]	87%
2. Are competing alternatives clearly described?	[3–25–26–27–28–29–30–31–32–33–36–37–38–39–40–41–42–43–44–45–46–47– 48–49–50–51–53–54]	97%
3. Is a well-defined research question posed in answerable form?	[3–25–26–27–28–29–30–31–32–33–36–37–38–39–40–41–42–43–44–45–46–47– 48–49–50–51–53–54]	97%
4. Is the economic study design appropriate to the stated objective?	[3–25–26–32–33–37–38–39–40–41–42–44–45–46–47–49–50–51]	63%
5. Is the chosen time horizon appropriate in order to include relevant costs and consequences?	[3–25–26–27–28–29–30–31–32–33–34–36–37–38–39–40–41–44–45–47–48–49–50–51–53–54]	90%
6. Is the actual perspective chosen appropriate?	[3–25–26–28–29–30–31–32–33–36–37–38–39–40–41–42–43–47–49–50–51–53–54]	80%
7. Are all important and relevant costs for each alternative identified?	[3–25–26–27–28–29–30–31–33–34–36–37–38–39–41–42–43–45–48–49–51–53–54]	60%
8. Are all costs measured appropriately in physical units?	[3–25–26–28–30–31–32–33–36–37–38–39–40–41–42–43–45––47– 48–49–50–51–53–54]	80%
9. Are costs valued appropriately?	[3–26–29–30–31–32–33–36–37–39–46–47–50–53–54]	83%
10. Are all important and relevant outcomes for each alternative identified?	[3–29–30–31–32–33–36–37–39–46–47–50–53–54]	53%
11. Are all outcomes measured appropriately?	[3–26–27–28–30–31–33–34–36–37–39–40–41–42–45–46–50–53–54]	67%
12. Are outcomes valued appropriately?	[3–26–28–29–30–31–32–33–36–37–39–40–41–42–45–46–47–50–53–54]	70%
13. Is an incremental analysis of costs and outcomes of alternatives performed?	[3–26–29–32–37–39–40–41–42–45–47–50–54]	43%
14. Are all future costs and outcomes discounted appropriately?	[3–26–27–28–29–31–32–33–36–38–39–40–41–42–43–45–47–48–49–50–53–54]	76%
15. Are all important variables, whose values are uncertain, appropriately subjected to sensitivity analysis?	[27–28–33–36–37–38–39–40–41–42–47–49–50–54]	50%
16. Do the conclusions follow from the data reported?	[3–25–26–27–28–29–30–31–32–33–34–36–37–38–39–40–41–42–43–44–45–46–47–48–49–50–51–53–54]	100%
17. Does the study discuss the generalizability of the results to other settings and patient/client groups?	[3–25–27–28–29–30–31–32–33–34–36–37–38–40–41–42–44–45–46–47–50–53–54]	80%
18. Does the article indicate that there is no potential conflict of interest of study researcher(s) and funder(s)?	[3–26–27–30–31–32–34–37–38–39–40–41–42–44–45–46–47–49–50–51–53–54]	73%
19. Are ethical and distributional issues discussed appropriately?	[3–27–30–31–33–35–36–37–44–46–53–54]	43%

### Cost categories

Most of the studies (n = 26) examined considered *direct costs* (see **Table 1**). These studies reported a diverse range of direct costs: costs for inpatient (costs of general and psychiatric hospital services) and outpatient treatment, rehabilitation, medication or emergency room treatments were assessed by most evaluations.

Five of the studies in this review presented data on both direct and indirect costs [3,31,45,52]. *Indirect costs* items included mortality, early retirement, sickness absence, production losses, work disability and reduced quality of life. One study [38] focussed exclusively on indirect costs, no direct costs were examined.

*Intervention* related costs were reported by 16 studies (see **Table 1**: Intervention Costs)

Three studies included in this review did not report any data on direct or indirect cost [27,44,46]. Goodman et al. (2011) [27] focused on the out-of-pocket expenses and insurance costs directly related to the treatment of BPD (i.e. therefore categorised as intervention costs in this review). In addition, Prendergast & McCausland (2007) [44] and Richter and colleagues (2013) [46] did not provide the actual (in)direct costs for inpatient and/or outpatient treatment in the paper, only cost-savings of providing DBT compared to TAU were provided (i.e. cost offset PT vs. TAU).

### Cost outcome approach

The majority of studies (18 evaluations) presented data on *post- intervention costs* only [27,32,33,38–51]. Eleven studies presented data on healthcare costs before (*pre*) and after (*post*) providing the intervention [26,28–31,34,36,37,44,53,54]. One study reported only on *pre-intervention costs* [52]. See **Table 1**.

### Results of the economic evaluations

#### Primary analysis

The calculated cost offsets associated with the provision of (one or two competing) psychotherapeutic interventions (PT) (i.e. Cost offset PT) and the additional cost-savings of implementing evidence-based psychotherapy compared to TAU (i.e. Cost offset PT vs. TAU) per patient per year, for each treatment approach separately, is shown in Table 1. Three studies [3,27,52] only provided data on costs either before or after the intervention; therefore no cost-savings comparison (i.e. cost- off set calculation) could be made. In addition, we did not attempt to provide the overall cost savings of TAU. Only two studies included in this review reported on pre- and post- cost data associated with the provision of TAU [25,26]. However, these findings are not sufficient to offset to any extent the cost-savings of the provision of TAU in the treatment of patients with BPD. Therefore TAU-related cost-data was not reported.

#### Cost offset PT

All studies comparing cost pre- and post intervention (15 evaluations; total sample size n = 787) reported a cost saving found to be in the range from USD $4 to $56,024 per person/per year. The pre-post analysis resulted in a mean weighted cost-saving of USD $5,840.92 [SD = $10,816.56; SE = $1,479.57] per patient per year (see Total **Table 1** Cost Offset PT). Only the study by Palmer and colleagues (2006) [26] reported an increase of $99 in costs per patient/per year, post- intervention.

Because single group pre-post tests have recognised limitations, we have run additional analysis without the non-controlled studies to overcome bias of our results. Seven non- controlled studies were identified as so [28–31,34,36,54] with a total sample size of n = 192. For these non-controlled studies, the pre-post analysis resulted in a mean weighted cost-saving of USD $14,682.56 [SD = $ 1,286.07; SE = $17,820.38] per patient per year.

Subsequenly, re-running our primary pre-post analysis of the costs of psychotherapeutic interventions (cost offset PT) excluding the non-controlled studies yielded similar outcomes. Without the inclusion of these seven non-controlled studies the overall weighted cost offset of PT was: USD $2,987.82 [SD = $4390.31; SE = $180.01] per patient per year across these studies.

#### Cost offset PT vs. TAU

The 15 evaluations (total sample size n = 1,415) comparing the healthcare costs of psychotherapeutic interventions for BPD to TAU related costs, mostly reported cost-savings. See **Table 1** Cost Offset PT vs. TAU. Compared to treatment as usual, the additional weighted mean cost-savings of implementing evidence-based psychotherapy was USD $1,551.37 per person per year [SD = $6,574.17; SE = $174.77]. These cost-savings ranged from $83 to $29,392 per person/per year. Five evaluations [41,42,45,47,50] did not find a cost-saving and reported an increase in costs when comparing healthcare costs of psychotherapeutic interventions for BPD to TAU related costs (increase in costs ranged from $61 to $10,772). See **Table 1.**

#### Secondary analysis

We furthermore were interested in whether there were detectable differences in the cost-benefit of different types of psychological therapy. We were able to find six studies of DBT and seven non-DBT alternative approaches. There are not enough studies to directly compare two alternative approaches to date, we therefore pooled the seven non-DBT studies. DBT was compared to TAU in six evaluations [33,40–43,45]. The (weighted) mean cost-offset derived from the provision of DBT compared to TAU was USD $78.43 [SD = $3,1412.83; SE = $184.01] per patient per year across these studies (total sample size n = 344). Seven non-DBT evaluations focused on the post- healthcare costs related to the provision of other psychological approaches, namely: MBT [25,47]; CI [48]; CP [49]; CBT-PD [51]; CBT-TAU [26] and MACT [50]. Compared to TAU, a (weighted) mean increase in costs of USD -$1,150.03 [SD = $5,482.33; SE = $170.91] per patient per year was demonstrated across the studies (total sample size n = 1029).

The weighted mean difference in cost savings vs. TAU derived after the provision of DBT vs. non-DBT studies was: $1,228.43 (SE = $251.13; 95% confidence interval $735.63 to $1,721.30). This resulted in a significant weigthed mean difference (*t*(953.33.) = 4.892, p = .000). Although the difference per patient is significant, it is important to recognise that treatments themselves begin from different cost bases–and DBT standard includes weekly 2.5 hours of group, one hour of individual and ancillary care and phone coaching–which is generally more intensive that the comparative treatments which are typically up to two hours individual per week. In addition, crisis care, because it diverts from hospitalisation, has a large cost-offset. Although there are many potential interpretations for this significant finding, it could suggest that there is a greater cost-benefit of non-DBT vs DBT approaches compared to TAU. However, caution should be used when interpreting these results.

## Discussion

### Summary of main findings

This paper reviews data published in peer review journals on the economic evaluations of evidence-based treatments for BPD, to inform recommendations for current mental health care funding policy. The results indicate that providing evidence-based treatments for BPD is cost-effective and results in cost savings. Our 2015 US dollar cost-offset calculations indicated that each individual in BPD treatment showed a reduction of USD $2,988 per patient per year in total healthcare costs in the year following BPD treatment as compared to the year prior to BPD. Perhaps more importantly, this review demonstrated that compared to treatment as usual, that the provision of psychotherapy resulted in an additional cost-saving of USD $1,551 per patient per year.

Up to December 2015, thirty economic evaluations across 29 studies met the inclusion criteria of this review and provided valuable cost-data for calculating a cost-savings for the psychotherapies evaluated. Of the studies included in this review, fifteen evaluations examined the economic benefits of a psychotherapeutic intervention using pre- and/or post-measures, but without a control group. Another fifteen evaluations provided data on the use of mental health services and related costs of psychotherapeutic intervention compared to treatment as usual (TAU) or client centred therapy (CCT) as a control group. The costs data presented in three evaluations [3,27,52] could not be used to calculate cost-savings. However, because our aim was to include all original studies reporting cost or cost-effectiveness data of BPD and to reflect the financial burden associated with BPD, these studies were included in the review and used to maximise the available information about the economic value of the provision of psychological treatment of BPD. Examining the studies included, DBT was the most evaluated treatment for BPD (15 evaluations). Other psychotherapeutic interventions examined by the studies included in this review were CM (2 evaluations), MBT (2 evaluations), SFT (2 evaluations) and one each for CBT, CBT-PD CI, CP, LT-P, MACT, OPC, SCP, SDC and TFP, representing a variety of intervention approaches (see Table 2 for description of treatments). The mean cost-savings derived from the provision DBT compared to TAU, did differ significantly from the additional cost-savings as a result from other forms of psychotherapy (6 DBT vs. 7 non-DBT comparisons). These findings could suggest that there is a greater cost-benefit of non-DBT vs DBT approaches compared to TAU. However, this finding is exploratory in nature and should not be used to draw firm conclusion about the cost savings of DBT or other forms of psychotherapy as there are variations across studies in approach, setting, and context. Future studies should compare costs of alternative treatments within the same project under similar conditions.

Our results suggest that psychotherapy for borderline personality disorders, independent of the type of treatment, can lead to cost-savings. Comparison of the cost savings of DBT versus other forms of psychotherapies did not lead to a significant difference in cost reduction; strengthening the status of the use of any form of well evaluated psychological therapy as the main treatment of borderline personality disorders [9,55]. Our findings furthermore provide evidence to support the assertion that offering effective therapy in BPD generates cost-saving advantages in terms of both direct and indirect healthcare costs.

### Strengths and limitations

Other reviews that have looked at economic evaluations [17,18,56], similarly found evidence for cost-effective treatments for patients with BPD. To the best of our knowledge, there is no recent study that estimated annual net cost-savings of providing treatment in patients with BPD in a systematic way as conducted here. Brettschneider and colleagues (2014) [18] older study reviewed 15 economic evaluations of psychological therapies for BPD, concluding that the economic evidence is not sufficient to draw conclusions and current evidence should be interpreted with caution due to methodological shortcomings. Our review updated this previous work and now includes 30 evaluations, providing firmer evidence for cost benefit.

Our estimates for cost benefit should be interpreted with caution as there was significant variation between studies. However, as most studies included a control or active treatment comparison, our estimates do reflect the entire evidence we were able to systematically review.

The key strength of our review is that it has used full resource-use data that have been reported by researchers who have either conducted an economic evaluation of clinical trials or reported healthcare costs associated with the provision of treatment for BPD. Moreover, the focus of our review was not limited to specific psychological therapies and/or economic evaluations performed alongside clinical effectiveness randomized controlled trials. Hence, in this review, adjustment of the cost-data to account for variation in the cost-methods used in the different economic evaluations allowed comparison between treatments and enhanced generalizability of the study results.

However, this review was restricted to a certain extent by some limitations. We found that among the economic evaluation studies there was a great heterogeneity in the definition of costs, as have previous reviews [18]. Although we made a concerted effort to identify cost-categories, in some cases classifying the reported healthcare costs as either direct or indirect costs was not clearly and based upon common-sense reasoning. Furthermore, the studies in this review reported data from different countries, which could possible affect the generalisability of study findings. There are differences in economic circumstances and in health systems across various countries resulting in corresponding differences in health outcomes and their costs. For example, the health service and societal perspective in the Netherlands is different compared to the UK and Australian system. In the Australian, there is universal coverage for health care services; with the federal government paying a large part of the cost of health services, whereas Health insurance in the Netherlands is mandatory and everyone has to take out their own basic healthcare insurance [57]. Although we have made a considerable effort by calculating costs using purchasing power parity PPP, this should be taken into consideration when interpreting the results.

Despite the demonstrated overall cost savings across the majority of the 30 evaluations included, the cost-offset calculations for six economic evaluations included in this review did not result in cost-savings [26,41,42,45,47,50]. In four of these [41,42,47,50] it may be that the extra costs associated with the alternative therapy compared to the costs of the provision of TAU may be due to the method of cost modelling in the studies [17,18]. In some cases, resource use was estimated by authors by regression models [17], and for these economic evaluations costs may have been overestimated. Furthermore, the healthcare costs incurred before and after the provision of CBT [26] and after the provision of DBT [45] compared to TAU, did not result in cost-savings. However, the cost differences in both studies were small and did not approach conventional levels of statistical significance. In addition, it must be noted that the intervention (CBT) examined by Palmer and colleagues (2006) [26] did result in an overall saving of USD $131 (per patient per year) in healthcare costs when compared with the provision of TAU.

This systematic review aimed to provide an overview of the cost savings associated with the provision of psychotherapeutic interventions for the treatment of BPD and was not intended as a statement of the clinical effectiveness of these treatments. Data was extracted based on cost alone, without further consideration of clinical effectiveness of these treatments and is outside the scope of this review. However, the evidence on economic outcomesalone is one factor informing clinical decision making in health care. Future studies may investigate the interaction between cost and clinical improvement, although this will rely upon an even larger pool of studies of sufficient methodological rigour to allow such an evaluation.

Although we were able to identify thirty economic evaluations in total, a paucity of material is apparent in this field, especially if we compare the modest number of economic findings with the larger number of clinical evaluations. We have found some papers aiming to evaluate the clinical and cost-effectiveness of evidence-based BPD treatment [58–60]. However, since these papers only outline the background and methods of randomised controlled trials and are still ongoing, these studies may inform future reviews.

## Conclusions

Borderline Personality Disorder (BPD) is considered one of the most expensive mental disorders in terms of direct and indirect healthcare costs. Evidence-based treatment approaches for BPD are available and demonstrate potential cost-saving when implemented effectively. This review is the first to calculate the cost-savings associated with the provision of evidence-based treatment for patients with BPD using all published data available for review. Evidence for cost-effectiveness of psychological treatment was supported by our findings. Based on the findings of this systematic review, the provision of evidence-based psychological treatment to patients with BPD results in a reduction in costs associated with both the use of mental health services and related community costs, that significantly exceeds the cost of no treatment or treatment as usual.

## Supporting information

S1 Table PRISMA Checklist. (DOC)
